# Response of adult honey bees treated in larval stage with prochloraz to infection with *Nosema ceranae*

**DOI:** 10.7717/peerj.6325

**Published:** 2019-02-08

**Authors:** Uros Glavinic, Tanja Tesovnik, Jevrosima Stevanovic, Minja Zorc, Ivanka Cizelj, Zoran Stanimirovic, Mojca Narat

**Affiliations:** 1 Department of Biology, Faculty of Veterinary Medicine, University of Belgrade, Belgrade, Serbia; 2 Department of Animal Science, Biotechnical Faculty, University of Ljubljana, Ljubljana, Slovenia

**Keywords:** *Nosema*, Prochloraz, Gene expression, Immunity, *Apis mellifera*, Colony loss

## Abstract

Among numerous factors that contribute to honey bee colony losses and problems in beekeeping, pesticides and *Nosema ceranae* have been often reported. In contrast to insecticides, whose effects on bees have been widely studied, fungicides did not attract considerable attention. Prochloraz, an imidazole fungicide widely used in agriculture, was detected in honey and pollen stored inside hives and has been already proven to alter immune gene expression of honey bees at different developmental stages. The aim of this study was to simulate the realistic conditions of migratory beekeeping, where colonies, both uninfected and infected with *N. ceranae*, are frequently transported to the vicinity of crop fields treated with prochloraz. We investigated the combined effect of prochloraz and *N. ceranae* on honey bees that faced fungicide during the larval stage through food consumption and microsporidium infection afterwards. The most pronounced changes in gene expression were observed in newly emerged *Nosema*-free bees originating from colonies previously contaminated with prochloraz. As exclusively upregulation was registered, prochloraz alone most likely acts as a challenge that induces activation of immune pathways in newly emerged bees. The combination of both stressors (prochloraz and *Nosema* infection) exerted the greatest effect on six-day-old honey bees. Among ten genes with significantly altered expression, half were upregulated and half downregulated. *N. ceranae* as a sole stressor had the weakest effects on immune gene expression modulation with only three genes significantly dysregulated. In conclusion, food contaminated with prochloraz consumed in larval stage could present a threat to the development of immunity and detoxification mechanisms in honey bees.

## Introduction

The value of honey bees (*Apis mellifera*), as honey producers, is minor compared to their inestimable role in the pollination of crops and wild plants (De la Rua et al., 2009; Gallai et al., 2009; Moritz et al., 2010). Unfortunately, considerable losses of honey bee colonies have been reported in Europe and the United States, with no definitive explanation. As no single factor has been identified as primary cause of this phenomenon, scientific consensus is that chronic exposure to multiple, interacting, and sometimes synergistic stressors (microbial pathogens, parasites, pests, exposure to pesticides, loss of forage and incorrect beekeeping practices) underlies honey bee colony losses (Goulson et al., 2015; Neumann & Carreck, 2010; VanEngelsdorp & Meixner, 2010).

Pesticides used in agriculture indirectly contribute to colony collapses and bee declines by increasing negative effects of diseases and/or parasites (Sanchez-Bayo & Goka, 2014). The most common flowering crops protection products found in bees and bee products are fungicides (Johnson et al., 2013; Mullin et al., 2010). Prochloraz is an imidazole fungicide with an ergosterol-biosynthesis inhibiting (EBI) function that is widely used (Vinggaard et al., 2006) and found in honey and pollen stored inside hives (Lambert et al., 2013). Previous studies have shown that prochloraz, used alone and in combination with coumaphos, inhibited the detoxification activity of cytochrome P450 (Johnson et al., 2006) and altered immune gene expression in honey bees (Cizelj et al., 2016).

*Nosema ceranae* is highly prevalent endoparasite (Stevanovic et al., 2011; Vejnovic et al., 2017) that infects the midgut of adult honey bees (Fries, 2010), but it has been detected in other tissues (Chen et al., 2009; Copley & Jabaji, 2012; Gisder et al., 2010) and haemolymph (Glavinić et al., 2014) as well. It is considered a serious threat to beekeeping industry (Simeunovic et al., 2014a), especially in some regions where dramatic colony losses were clearly attributed exclusively to *N. ceranae* infections (Higes et al., 2008; Higes et al., 2009; Martin-Hernandez et al., 2007). *N. ceranae* and synergistic factors were reported as the cause of severe losses of honey bee colonies (Bacandritsos et al., 2010; Bromenshenk et al., 2010). *N. ceranae* was found to suppress the honey bee immune response (Antunez et al., 2009; Chaimanee et al., 2012), but stronger negative effects on bees were induced by *N. ceranae* infection in combination with exposure to pesticides (Aufauvre et al., 2012; Aufauvre et al., 2014; Vidau et al., 2011).

The aim of this study was to investigate the effects of fungicide consumption during the larval stage and microsporidium infection three days after emergence of adult honey bees. In a combination of field and laboratory experiment we tested our hypothesis that prochloraz from the environment may reach the larvae and disturb the immune response of newly emerged bees boosting the negative impact of *Nosema* infection that bees are faced with during the adult life. Our experimental approach assessed the most realistic situation of beekeeping in warmer climates, where colonies are moved to agricultural regions during the main season so their brood is exposed to agricultural pesticides, including prochloraz and majority of colonies are infected with *N. ceranae* (Bacandritsos et al., 2010; Higes et al., 2013; Stevanovic et al., 2016; Stevanovic et al., 2013).

## Material and Methods

### Field experiment

The experimental apiary was situated at Faculty of Veterinary medicine (FVM), University of Belgrade, Serbia. Healthy colonies headed by sister queens (*Apis mellifera*) without clinical signs of brood and adult bee disease were chosen for the experiment. The first colony was treated with prochloraz and was placed far away from the apiary to prevent chance of drifting. The second colony served as control. Honey reserves were completely removed from colonies. Each day during the whole month, treatment colony was fed with 200 ml of sugar syrup with prochloraz (Sigma, Darmstadt, Germany) in concentration 10 µg/kg, while the control received pure sugar syrup. Additionally, to affect honey bee larvae, pollen (bee bread) was contaminated by spraying with prochloraz dissolved in sugar syrup (in concentration 10 µg/kg). Prochloraz concentration used was in range detected in contaminated honey and pollen stored inside hives (Lambert et al., 2013).

### Laboratory experiment

A month after the first day of prochloraz treatment, one frame with sealed brood (prior to emergence) from treated and one from control colony were transferred to the Laboratory for Animal Genetics (at FVM, in close vicinity of the apiary). Frames with brood were kept in separated incubators (at 34  ± 1  °C) until bee emergence. At the time of emergence seven bees from each frame were collected for gene expression analysis, representing zero-day samples (C0 and P0). Newly emerged worker bees were removed from both frames and confined to cages designed by Glavinic et al. (2017). Each experimental group (Control, Prochloraz, *Nosema*, Prochloraz and *Nosema*) contained three cages with 30 bees per cage. One of three cages in each group was reserved for sampling on day six, second cage for day nine, and last one for day 15, as shown in Table 1.

**Table 1:** Experimental design and description of experimental groups.

Sampling^a^	Prochloraz non-treated groups		Prochloraz treated groups	
	Non-infected bees (C)	*N. ceranae* infected bees^b^ (CN)	Non-infected bees (P)	*N. ceranae* infected bees^b^ (PN)
day 0	C0^c^	/^d^	P0	/^d^
day 6	C6	CN6	P6	PN6
day 9	C9	CN9	P9	PN9
day 15	C15	CN15	P15	PN15

**Notes.**

a Number of days after emergence; on each sampling day seven honey bees were collected for qPCR gene expression analysis.

b Infection with *N. ceranae* was performed on 3rd day after honey bees emergence.

c Experimental group designation.

d Samples were not collected.

Fresh *N. ceranae* spore suspension with a minimum spore viability of 99% (assessed with 4% trypan blue) was mixed with 50% sucrose solution to obtain the inoculum with a final concentration of 1.000.000 spores/mL. Bees in six cages (groups CN6, CN9, CN15, PN6, PN9, and PN15) were infected on the third day after emergence as described by Fries et al. (2013). Other non-infected cages were fed with pure 50% sucrose solution. In all cages food was consumed readily without regurgitation.

### RNA isolation and cDNA synthesis

For gene expression analysis, seven bees from each group were collected on each sampling day (0, 6, 9 and 15 days after emergence). Each individual honey bee was placed in sterile 1.5 mL polypropylene microtube (Eppendorf) with 200 µL of lysis buffer (Zymo Research, Irvine, CA, USA) and homogenized with sterile disposable microtube pestles (VWR, San Francisco, CA, USA). The total RNA was isolated from individual sample using the Quick-RNA MiniPrep Kit (Zymo Research). Following to manufacturer’s instructions of Quick-RNA MiniPrep Kit the samples have passed through DNase treatment in order to remove any contaminating DNA. The extracted RNA was immediately used to generate cDNAs using the RevertAid™ First Strand cDNA Synthesis Kit (Fermentas, Waltham, MA, USA).

### Real-time qPCR

Primer pairs for 19 examined genes (15 immune related genes, two detoxification genes, and two housekeeping genes) were those reported in Gregorc et al. (2012), Tesovnik et al. (2017), and Evans (2006) (Table 2). For quantitative real-time PCR (RT q-PCR), 10 µL reactions were prepared, containing 5 µL of Fast Start Universal SYBR Green Master (ROX) (Roche Diagnostics GmbH, Germany), 250 nM of forward and reverse primer, DEPC treated water and 1µL of cDNA (5 ng per reaction). Amplification of targeted molecules was performed with ViiA7 (Applied Biosystems, Foster City, USA) and analysed with QuantStudio™ Real-Time PCR Software. For the experimental run the following cycle profile was used: denaturation step at 95 °C for 10 min, and 40 cycles at 95 °C for 20 s, 20 s at Tm of each primer pair and 72 °C for 20 s, followed by dissociation curve step at 95 °C for 15 s, 60 s at Tm of each primer pair and 95 °C for 15 s, when temperature was gradually rising from Tm to 95 °C by 0.5 °C increments per cycle. Reactions for RT q-PCR were carried out in 384-well plates (MicroAmp^®^; Life Technologies). Each run contained three no-template controls and test samples preformed in duplicates. Gene expression was analysed for 15 immune-related genes and two detoxification genes. We tested the set of candidate normalization genes (*actin* and *RPS5*) as possible housekeeping genes. A geNorm algorithm-based analysis (Vandesompele et al., 2002) indicated *RPS5* as the most suitable housekeeping gene. Gene expression values of non-treated group were used for gene expression calibration. For each gene the level of gene expression was calculated using the method described by Pfaffl (2001), where the relative expression ratio between treated and non-treated group is based on PCR efficiency. These results were then visualized on a heatmap illustrating expression of genes as a consequence of different treatments. The significance was indicated according to the statistics described below.

All collected samples were also tested for the most common honey bee pathogens using RT q-PCR as described above (Table 2). Tested samples were positive only for *N. ceranae* and Deformed wing virus (DWV) and its RNA loads were evaluated by comparing threshold cycle (Cq) values between treatment groups (Badaoui et al., 2017; Cizelj et al., 2016; Zheng et al., 2015).

**Table 2:** Primers used in this study.

Targets/Locus gene ID	Gene description	Sequences of primers used in qPCR	Efficiency (%)	*R*^**2**^
***Pathogen targets***				
*A. apis* AY004344	*Ascosphaera apis* 28S large subunit ribosomal RNA gene	F: TCTGGCGGCCGGTTAAAGGCTTC R: GTTTCAAGACGGGCCACAAAC	*NA*	*NA*
*A. woodi* HQ243442.1	*Acarapis externus* isolate B4E5 cytochrome oxidase subunit I	F: TCAATTTCAGCCTTTTATTCAAGA R: AAAACATAATGAAAATGAGCTACAA	*NA*	*NA*
ABPV HM228893.1	Acute bee paralysis virus isolate GFf1ab	F: ACCGACAAAGGGTATGATGC R: CTTGAGTTTGCGGTGTTCCT	*NA*	*NA*
BQCV HQ655494.1	Black queen cell virus	F: TTTAGAGCGAATTCGGAAACA R: GGCGTACCGATAAAGATGGA	*NA*	*NA*
DWV AY292384.1	Deformed wing virus isolate	F: GAGATTGAAGCGCATGAACA R: TGAATTCAGTGTCGCCCATA	99.3	0.981
IAPV EU224279	Israel acute paralysis virus of bees	F: GCGGAGAATATAAGGCTCAG R: CTTGCAAGATAAGAAAGGGGG	*NA*	*NA*
KBV AY275710.1	Kashmir bee virus	F: TGAACGTCGACCTATTGAAAAA R: TCGATTTTCCATCAAATGAGC	*NA*	*NA*
*P. larvae* DQ811780.1	*Paenibacillus larvae*	F: CGGGAGATGAGAAAACCAAT R: CCGCAATCGTAAGCTGGTAT	*NA*	*NA*
***Housekeeping genes***				
Actin GB44311	Actin - cytoskeletal structural protein	F: TTGTATGCCAACACTGTCCTTT R: TGGCGCGATGATCTTAATTT	98.1	0.996
RPS5 GB11132	Ribosomal protein S5a	F: AATTATTTGGTCGCTGGAATTG R: TAACGTCCAGCAGAATGTGGTA	101.0	0.989
***Immune related genes***				
Abaecin GB18323	Abaecin, antimicrobial peptide	F: CAGCATTCGCATACGTACCA R: GACCAGGAAACGTTGGAAAC	104.3	0.992
Basket GB56012	JNK MAP kinase	F: AGGAGAACGTGGACATTTGG R: AATCCGATGGAAACAGAACG	96.7	0.992
Cactus GB19910	IkB transcription factor	F: CCTGGACTGTCTGGATGGTT R: TGGCAAACCCTTTCTCAATC	98.8	0.979
Defensin-1 GB41428	Defensin 1	F: TGCGCTGCTAACTGTCTCAG R: AATGGCACTTAACCGAAACG	101.0	0.983
Defensin-2 GB10036	Defensin 2	F: GCAACTACCGCCTTTACGTC R: GGGTAACGTGCGACGTTTTA	96.4	0.992
Domeless GB16422	Cytokine receptor; JAK-STAT immune signalling pathway	F: TTGTGCTCCTGAAAATGCTG R: AACCTCCAAATCGCTCTGTG	104.1	0.997
Dorsal-1 GB19537	NFkB transcription factor orthologue	F: AGAGATGGAACGCAGGAAAC R: TGACAGGATATAGGACGAGGTAA	98.7	0.994
Hopscotch GB44594	JAK tyrosine kinase	F: ATTCATGGCATCGTGAACAA R: CTGTGGTGGAGTTGTTGGTG	103.2	0.995
Kayak GB12212	Fos, the Drosophila homologue of the mammalian proto-oncogene product c-Fos	F: CGACAGATCCGCAGAGAAAG R: CCTGTTGCAGCTGTTGTATC	98.0	0.988
Lys2 GB15106	Lysozyme; immune system-end product	F: CCAAATTAACAGCGCCAAGT R: GCAATTCTTCACCCAACCAT	102.4	0.994
PGRPSC4300 GB15371	Peptidoglycan recognition protein S1	F: GAGGCTGGTACGACATTGGT R: TTATAACCAGGTGCGTGTGC	104.6	0.996
Spaetzle GB15688	Toll-binding cytokine-like molecule	F: TGCACAAATTGTTTTTCCTGA R: GTCGTCCATGAAATCGATCC	101.1	0.973
Toll GB18520	Toll-like receptor	F: TAGAGTGGCGCATTGTCAAG R: ATCGCAATTTGTCCCAAAAC	100.1	0.987
***Detoxification related genes***				
PKA-C1	cAMP-dependent protein kinase 1	F: TCCATTTTTGGTCTCCTTGC R: GTAAAAGCGCGAATGTGGTT	98.1	0.998
PKA-R1	cAMP-dependent protein kinase type I regulatory subunit	F: GAAGCAATTATTCGGCAAGG R: TCACCGAAACTTCCACCTTC	99.3	0.992

### Statistical analysis

**Figure 1:**
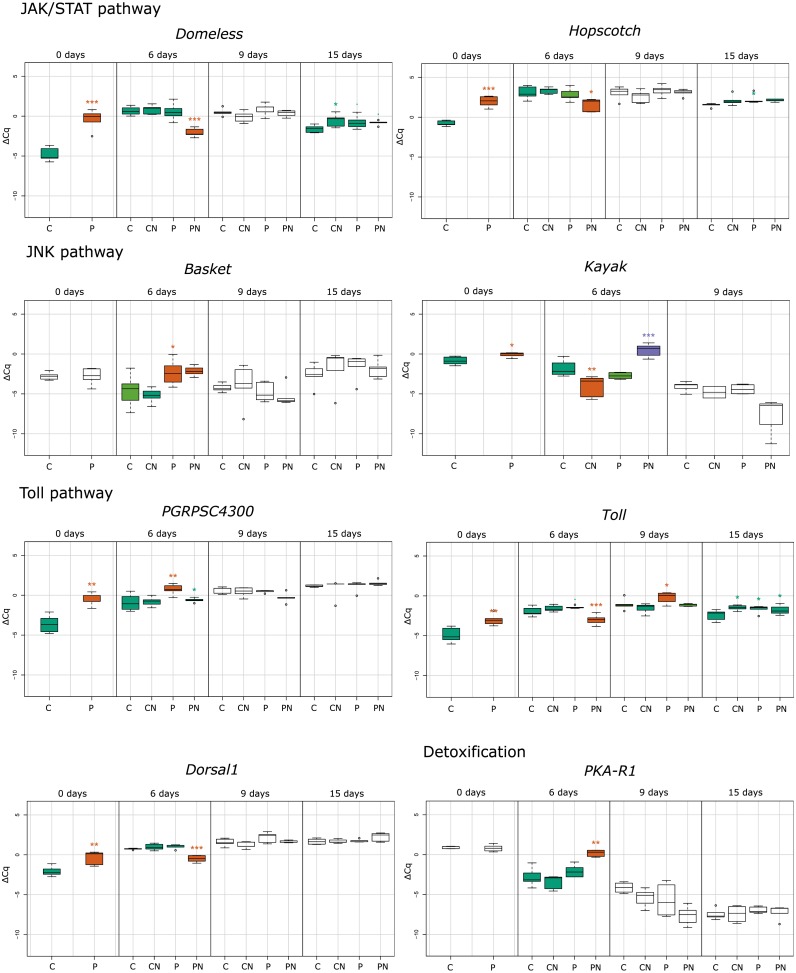
Treatment depended statistical analysis box plot diagram for immune–related and detoxification gene expression. Each box plot represents the ΔCq values measured for biological replicates for selected treatment. Boxes marked with an asterisk show statistically significant effects of treatment on gene expression when the *p*-value was equal or less than 0.05. If there were no significant differences among the groups, they share the same color. Treatments are indicated in the scale at the bottom of the plots (Control, C; *Nosema*-infected, CN; Prochloraz-treated, P; Prochloraz treated and *Nosema*-infected, PN). Analysis was undertaken with program R.

**Figure 2:**
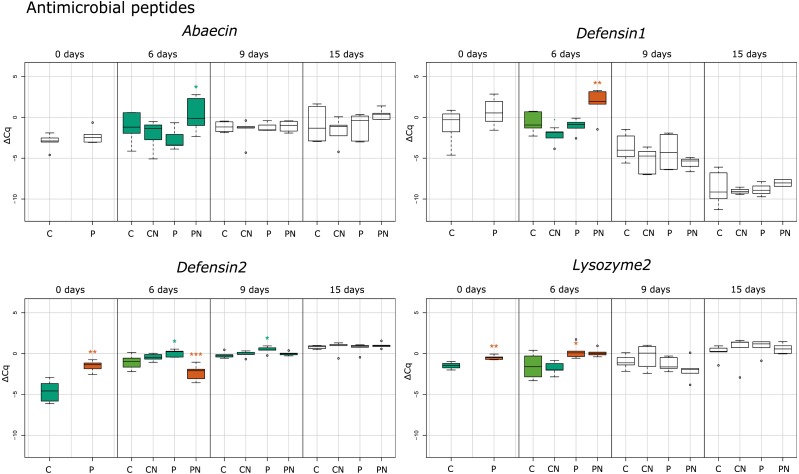
Treatment depended statistical analysis box plot diagram for gene expression of antimicrobial peptides. Each box plot represents the ΔCq values measured for biological replicates for selected treatment. Boxes marked with an asterisk show statistically significant effects of treatment on gene expression when the *p*-value was equal or less than 0.05. If there were no significant differences among the groups, they share the same color. Treatments are indicated in the scale at the bottom of the plots (Control, C; *Nosema*-infected, CN; Prochloraz-treated, P; Prochloraz treated and *Nosema*-infected, PN). Analysis was undertaken with program R.

All statistical analyses and plotting were performed using R software version 3.5.1 (R Core Team, 2017) with relevant libraries (lsmeans, moments, ggplot2) (Komsta & Novomestky, 2015; Lenth, 2016; Wickham, 2009). Relative expression levels of studied genes were normalized with housekeeping gene *RPS5*. Delta Cq (ΔCq) between housekeeping gene Cq values and target genes Cq values were calculated. To analyze the effects of *Nosema* infection, prochloraz treatment and interaction of both treatments on gene expression, we used a linear model for fixed effects (lm function in R) for each of 17 genes and each sampling group (0, 6, 9, 15 days after emergence) according to the following model (1):

(1) \begin{eqnarray*}{y}_{ijk}=\mu +{N}_{i}+{P}_{j}+{N}_{i}{P}_{j}+{e}_{ijk}\end{eqnarray*}

where *y*_*ijk*_ is ΔCq value, µ is overall mean, *N*_*i*_ is fixed effect of *Nosema* infection (i = yes, no), *P*_*j*_ is fixed effect of prochloraz treatment (j = yes, no) and *e*_*ijk*_ is residual error. The estimation of least squares means followed by Dunnett’s post hoc test was used for pairwise comparisons among the treatment groups. The assumption of normal distribution was tested and met via examination of the residuals (coefficients of skewness and kurtosis). The gene expression data (ΔCq values) and the results of statistical analysis were then graphically summarized using boxplots (Figs. 1 and 2) where treatments with statistically significant effect on gene expression were marked with an asterisk. If there were no significant differences among the groups according to post-hoc pairwise comparison test, they share the same color. A *p*-value less than 0.05 was considered statistically significant.

## Results

### Effects of larval prochloraz consumption on immune system gene expression of adult honey bees

**Figure 3:**
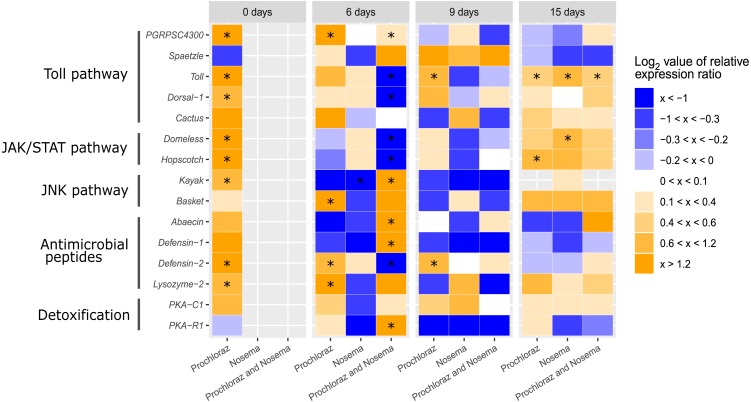
Heatmap immune-related genes in adult honey bee at different ages (0-, 6-, 9- and 15-days after honey bee emergence). The colors indicate the average mRNA levels compared to average levels of mRNA in control groups: blue indicate lower and yellow higher levels. Range log_2_ value of relative expression ratio is indicated in the legend on the right. Each row corresponds to one gene transcript and each column, to the expression profile of treatment. The gene names and corresponding pathway are indicated on left side. Treatments are indicated in the scale at the bottom of the graph (*Nosema*-infected, CN; Prochloraz-treated, P; Prochloraz treated and *Nosema*-infected, PN). Control group (C) was used for normalization. Boxes marked with an asterisk show statistically significant effects of the treatment on gene expression, when *p*-value was equal or less than 0.05.

In response to consumption of prochloraz in larval developmental stage (group P) the majority of genes involved in immune response were upregulated compared to control group (group C) in newly emerged honey bees (day 0). The most upregulated genes were the gene encoding cytokine receptor Domeless (4.39; *p* < 0.001), the gene encoding pathogen recognition protein PGRP-SC 4300 (3.04; *p* < 0.01), antimicrobial peptide (AMP) gene *defensin-2* (2.95; *p* < 0.001), and the gene for the JAK tyrosine kinase *hopscotch* (2.73; *p* < 0.001). Significant upregulation of gene expression was also noticed for genes encoding Toll (1.88; *p* < 0.01), Dorsal-1 (1.17; *p* < 0.01), Kayak (0.80; *p* < 0.05) and AMP Lysozyme-2 (0.88; *p* < 0.01). On day six after emergence genes *basket* (2.23; *p* < 0.05), *PGRPSC 4300* (1.68; *p* < 0.01), *lysozyme-2* (1.58; *p* < 0.05) and *defensin-2* (0.89; *p* < 0.05) were significantly upregulated in group P compared to group C. On 9th day in group P (compared to group C), significant increase in expression of *defensin-2* (0.61; *p* < 0.05) and *toll* (0.82; *p* < 0.05) was noticed and on 15th day the *hopscotch* (0.72; *p* < 0.05) and *toll* (0.50; *p* < 0.05) were significantly upregulated (Figs. 1–3).

### Effects of *Nosema* infection on gene expression of adult honey bees

The alterations in gene expression between *Nosema*-infected (group CN) and non-infected (group C) adult honey bees were significant only in three cases: significant downregulation of *kayak* gene (−2.02; *p* < 0.01) on day six after emergence and significant upregulation of genes *domeless* and *toll* on 15th day (0.78; *p* < 0.05 and 0.70; *p* < 0.05, respectively) (Figs. 1–3).

### Effects of larval prochloraz consumption on gene expression of adult honey bees infected with *Nosema*

In response to *Nosema* infection, the expression patterns of detoxification and immune-related genes differed between adult honey bees experienced prochloraz treatment during larval stage (group PN) and those from non-treated (group CN) colonies. The most significant changes were noticed on 3rd day after *Nosema* infection (on day six after emergence). The expression of gene *kayak* (1.97/−2.02), gene encoding AMPs Defensin-1 (2.34/−1.64) and gene *PKA R1* (2.49/−1.03) was significantly higher in group PN than in group CN. Conversely, transcript levels of genes involved in JAK/STAT immune pathway (*domeless*, −2.77/0.17 and *hopscotch*, −1.54/0.27), AMP gene *defensin-2* (−1.34/0.40) and genes involved in Toll immune pathway *dorsal-1* (−1.21/0.20), *toll* (−1.01/0.34) and *PGRPSC 4300* (0.31/0.10) were significantly lower in PN than in CN group. On day nine after emergence, the alternations in gene expression between prochloraz-treated and non-treated honey bees infected with *Nosema* were not significant, and on 15th day only the *toll* gene (0.42/0.70) was significantly downregulated (Fig. 3).

When gene expression levels of bees from PN group were compared to control bees (C group), in six-day-old honey bees from group PN, five immune-related genes were significantly downregulated: *domeless* (−2.77; *p* < 0.001), *defensin-2* (−1.34; *p* < 0.001), *dorsal-1* (−1.21; *p* < 0.001), *hopscotch* (−1.54; *p* < 0.05) and *toll* (−1.01; *p* < 0.001). Conversely, the expression of detoxification gene *PKA R1* (2.49; *p* < 0.01) and AMPs gene *defensin-1* (2.34; *p* < 0.01) and *kayak* (1.97; *p* < 0.001) was increased. In 15-day-old honey bees significant upregulation of only *toll* gene (0.42; *p* < 0.05) was recorded (Figs. 1 and 2).

### Effects of *N. ceranae* and prochloraz treatment on levels of Deformed wing virus (DWV) RNA load

**Figure 4:**
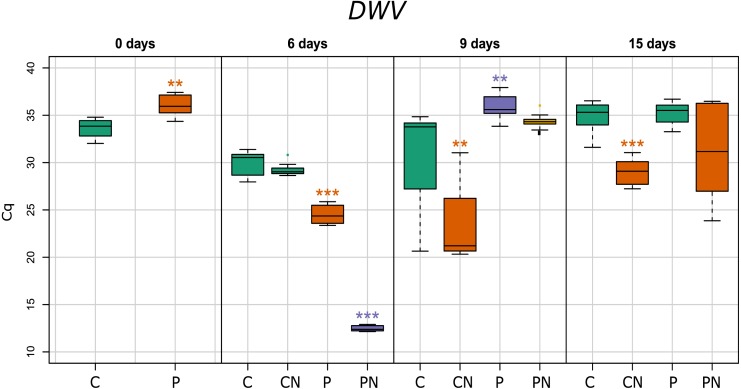
Deformed Wings Virus RNA loads in honey bees. Cq: qPCR signal for pathogen loads.

In *Nosema*-infected groups DWV RNA loads significantly increased on 6th and 9th day after *Nosema* infection. Prochloraz larval treatment decreased DWV RNA loads in newly emerged and nine-day-old honey bees and increased it on six-day-old honey bees. The most significant increase of DWV RNA loads was noticed in prochloraz affected and *Nosema*-infected six-day-old honey bees (Fig. 4).

### Effects of treatments on *Nosema* level

In both *Nosema* challenged groups, prochloraz-treated and non-treated, increase of *Nosema* RNA level was evident on day six and 15 of the experiment. However, *Nosema* level was higher in prochloraz-treated group (PN) than in prochloraz non-treated group (CN) (Fig. 1).

## Discussion

In this study the effects of two stressors, fungicide (prochloraz) and endoparasite (microsporidium *N. ceranae*), on adult honey bees were investigated for the first time. By keeping 30 bees per cage until sampling we assured that their social life disturbance could not have an effect on gene expression. The *Nosema* RNA level increased during the experiment in both *Nosema*-infected groups (CN and PN) as in our previous study (Glavinic et al., 2017). It is interesting that bees affected with both stressors (fungicide and *Nosema*) had higher *Nosema* levels than those that were only *Nosema*-infected (Fig. 1). This finding led us to suggest that prochloraz, being ingested by larvae via food, could reach the gut of newly emerged bees and could intensify *Nosema* infection.

Unlike previous finding of host immune suppression by *N. ceranae* infection (Antunez et al., 2009; Chaimanee et al., 2012; Glavinic et al., 2017), it seems that this study revealed only weak reaction to this parasite as the sole stressor. Only the gene *kayak* involved in JUN NH2-terminal kinase pathway was significantly downregulated (*p* < 0.01) in six-day-old honey bees. In 15-day-old bees expression of genes *domeless* and *toll* was upregulated (*p* < 0.05). Nevertheless, with the exception of *abaecin* and *defensin*-*1*, genes analysed in this study have not been monitored in previous investigations of *Nosema* influence (Antunez et al., 2009; Chaimanee et al., 2012; Glavinic et al., 2017). *Abaecin* was not downregulated in both this and our previous study (Antunez et al., 2009; Chaimanee et al., 2012; Glavinic et al., 2017), and *defensin-1* was suppressed in this study but not significantly, therefore no clear contrast was observed between current and other available data (Antunez et al., 2009; Chaimanee et al., 2012; Glavinic et al., 2017).

The effect of prochloraz on honey bee immune-related genes was estimated simulating the realistic field event of honey bee contamination (Lambert et al., 2013). As adult bees subjected to contamination do not live long, we hypothesize that pesticide contamination of honey bee colony has greater consequences for the honey bee brood, especially larvae that possibly receive pesticide through feeding by nurse bees. The results revealed the most pronounced changes in gene expression in newly emerged (*Nosema*-free) bees originating from colonies previously contaminated with prochloraz. In bees sampled on the day of emergence (day 0), we registered significant upregulation in eight genes out of 15 analysed, with most upregulated genes involved in JAK/STAT pathway *domeless* and *hopscotch* (*p* < 0.001) (Fig. 1). Other upregulated genes were genes involved in Toll-related immune pathway *PGRP-SC 4300*, *toll* and *dorsal-1*, genes encoding antimicrobial peptides (AMP) Defensin-2 and Lysozyme-2 (*p* < 0.01) and gene encoding Kayak protein involved in JNK pathway (*p* < 0.05). The results suggest that prochloraz consumed by adult nurse bees reached the larvae and affected all important pathways and mechanisms in charge for the honey bee self-defence in this early stage of new born bees. As exclusively upregulation was registered, we could propose that prochloraz contamination acts as a challenge that induces immune pathways activation in newly emerged bees. Although the reaction of immune genes varied during time, significantly changed genes were always upregulated in bees affected by prochloraz treatment. This data support findings in our previous study (Cizelj et al., 2016).

The effect of both stressors (prochloraz and *Nosema*) was most pronounced on six-day-old honey bees (three days after *Nosema* infection) in which ten genes had significantly changed expression. Five genes were upregulated with the greatest increase of expression recorded in *Kayak* (*p* < 0.001), followed by *Defensin-1* and detoxification related gene *PKA-R1* (*p* < 0.01), *abaecin* and *PGRPSC-4300* (*p* < 0.05). Among five genes that were downregulated, the decrease at level *p* < 0.001 was evidenced in four (*defensin-2*, *domeless*, *dorsal-1* and *toll*), and only one gene (*hopscotch*) had the decrease at level *p* < 0.05. In contrast, significant increase in the expression of the same genes (*p* < 0.001 for *domeless* and *hopscotch; p* < 0.01 for *defensin-2* and *dorsal-1*) was recorded in six-day-old bees challenged by prochloraz. It is interesting that no gene was significantly affected by the combination of prochloraz and *Nosema* infection in nine-day-old bees, while in 15-day-old bees only *toll* gene was significantly upregulated compared to control (*p* < 0.05). In the oldest bees significant upregulation (*p* < 0.05) of the *toll* gene was also induced by prochloraz treatment or *Nosema* infection alone. However, extremely opposite reaction of the same gene (significant downregulation at level *p* < 0.001) was recorded in six-day-old bees challenged with both prochloraz and *Nosema* infection. The absence of significant changes in expression of majority genes by both stressors on day 15 is possible when fungicide treatment and parasite infection (Fig. 1) have synergistic negative effect on honey bee health. But this synergism has not been proven on transcription level of immune genes we monitored. Elucidation of the mechanism that underlies the observed finding is required, particularly because of already proven negative effects of *Nosema* and pesticide combinations (Alaux et al., 2010; Aufauvre et al., 2012; Aufauvre et al., 2014; Vidau et al., 2011).

The colonies used in this study were positive for DWV, which is expected since the presence of this virus was previously reported in majority of Serbian honey bee colonies (Simeunovic et al., 2014b; Cirkovic et al., 2018). The synergistic effect between DWV and *N. ceranae* was investigated before (Costa et al., 2011; Doublet et al., 2015; Martin et al., 2013) but no consistent conclusion was made. In our study significant increase of DWV loads on 6th and 9th day after *Nosema* infection was recorded similar as in study of Zheng et al. (2015). Furthermore, the highest DWV load was noticed in prochloraz-challenged *Nosema*-infected six-day-old honey bees (Fig. 3). Prochloraz stimulating influence on DWV was noticed also in non-infected six-day-old honey bee while in non-infected newly emerged and non-infected nine-day-old honey bees, prochloraz decreased DWV loads. The stimulating effect of pesticide on DWV was noticed also in our previous study (Tesovnik et al., 2017).

## Conclusions

Overall, the results of this study confirm our hypothesis that honey bee food contamination with prochloraz presents the threat to the next generation of bees. Although we did not investigate how much larvae are contaminated, its contamination could be more important as they are affected in sensitive stage of development. The worst consequences could be presumed for colonies contaminated with prochloraz during late summer as their larvae develop into winter bees responsible for colony survival till spring. This scenario is likely to happen when beekeepers migrate their hives to sunflower forage that is in many regions the last in the season, so the bees are wintered on sunflower food. As sunflower fields are most likely contaminated with agricultural pesticides, there is great probability of sunflower contamination and consequently the brood intended to produce the population of winter bees. However, further investigations are required to reveal how the transcriptional disturbances in bee larvae during late summer influence winter survival of the colony.

##  Supplemental Information

10.7717/peerj.6325/supp-1 Data S1 Raw Data Raw data: Ct values of qPCR for tested genes.

10.7717/peerj.6325/supp-2 Data S2 Raw data Raw data: Statistical results of analysed data.

10.7717/peerj.6325/supp-3 Figure S1 *Nosema* RNA loads 6-, 9- and 15-days after bee emergence in *Nosema* (CN) and prochloraz treated and *Nosema* infected (PN) experimental groups

## Additional Information and Declarations

### Data Availability

The following information was supplied regarding data availability: Raw data is available in the Supplemental File.
