# Supplementary figures and images for: Response of adult honey bees treated in larval stage with prochloraz to infection with Nosema ceranae
Source: PeerJ. 2019 Feb 8;7:e6325. doi: 10.7717/peerj.6325 (PMC6371917; doi:10.7717/peerj.6325)

# *Nosema*

**6 days**

**9 days**

**15 days**

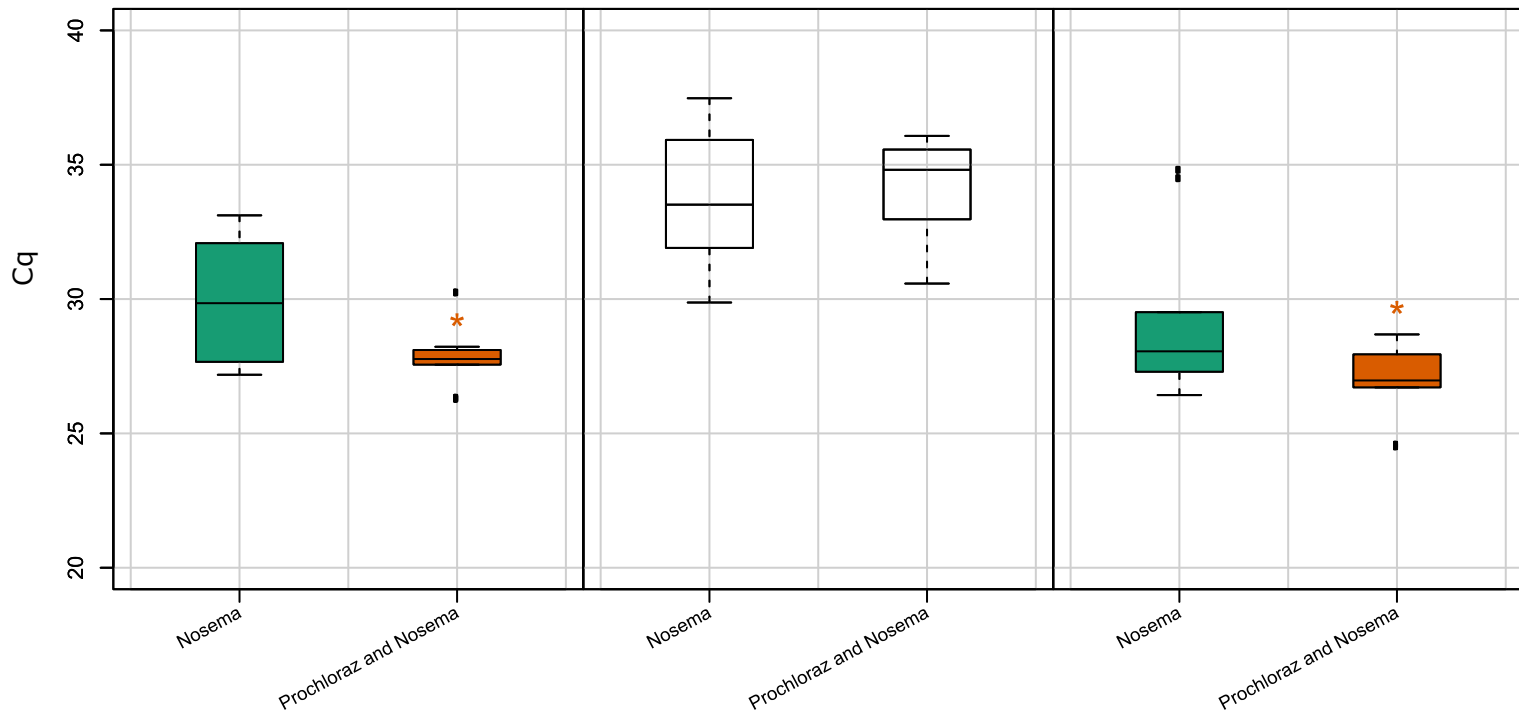

Supplement: Figure S1 [file peerj-07-6325-s003.pdf]
